# Practical Chemistry and Pharmacy

**Published:** 1894-08

**Authors:** 

**Affiliations:** LaGrange, Ga.


### PRACTICAL CHEMISTRY AND PHARMACY.

EDITED BY HENRY R. SLACK, M.D., PH.M., LAGRANGE, GA.

### PASKOLA.

Dr. Robert G. Eccles, of Brooklyn, contributes to the *Druggists' Circular* quite an interesting article on this much-advertised Predigested Food, from which we take the following extracts:

"The literature of this company contains some new theories in physiology. Take for example the following:

"'Paskola would make you fat if you had no stomach.'

"'Paskola contains a vegetable trypsin ferment which converts the albumen into peptones in the stomach.'

"'No stomach will digest uncooked starchy food.'

"'Paskola saves your digestive organs from having to work. When it reaches the intestines it is absorbed at once.'"

I would like to know how a man devoid of a stomach could be made fat by this preparation. It has been my idea hitherto that trypsin did work in an alkaline medium, while that of the stomach is acid. My impression has been that human stomachs do not digest either cooked or uncooked starch foods. They might digest the albuminous parts, but not the starch of so-called starchy foods.

The pamphlets further say: "Albumens are not fattening. If they don't agree with you, don't eat too much of them. Then you won't need pepsin." When the living being is evolved that albumens do not agree with, he will surely be a curiosity. Had we been advised not to eat too much albumen, such advice would have been good. An excess of anything is bad. One would almost think that they considered albumen as something that could be readily dispensed with, instead of being the very basis of life itself. Indeed they elsewhere tell us that it is "the least important of the foods we digest."

In an analysis of Paskola made by me I found it to be predigested starch (more commonly called glucose), but sought in vain for any natural vegetable ferments.

Paskola can be practically duplicated by the following formula:

Glucose syrup, 1 pound.

Hydrochloric acid, 50 drops.

Sulphurous acid U. S. P. (freshly prepared), 6 to 8 drops.

The public has a decided bias against the use of predigested starch under its proper name, glucose. This aversion has a rational basis, although with some it is excessive. In diabetes the naturally digested starch does exactly what it is claimed all predigested starch is sure to do. It goes immediately and without modification into the blood. To predigest starch would seem, therefore, to encourage diabetes, one of the most insidious diseases known to medical science; and I am not satisfied that it is "the outcome of the most modern discoveries of modern science," as is declared by its makers.

[I have also made an analysis of Paskola, and find the formulas as given by Dr. Eccles substantially correct..—H. R. S.]

---

### IMPROVED IODINE OINTMENT.

At the recent semi-annual meeting of the California Pharmaceutical Society, S. A. McDonnell reported that the following is a better way of preparing iodine ointment than the official method. Take four parts of iodine and ninety-six of lard. Melt the lard on a water bath, drop in the iodine, and stir with a glass rod until dissolved. The heat is not high, lard melts at about 35° C., (95°F.); and this low heat does not vaporize the iodine to any more appreciable extent than ordinarily, since it is only slowly volatilized at ordinary temperatures, and it requires 114° C. (237.2° F.) to melt it. Iodine ointment prepared in the manner described is superior, inasmuch as it is free from the hard crystals of potassium iodine which remain when the water has evaporated from the ointment, and thus avoid the scratching of tender skin caused by them on inunction.—*M. M. Rep. and Phar. Journal.*

---

### CHLORINE WATER IN DIPHTHERIA.

Dr. Schubert reports on the value of chlorine water in diphtheria. After an experience of many years he is most enthusiastic on the subject, claiming that no known treatment equals that of internal administration of chlorine water.

He gives a teaspoonful of a mixture consisting of two parts of chlorine water, and one part of distilled water, repeating every two or three hours according to the gravity of the attack. On account of the pungent odor the nose may be held for smaller children. Gargling or painting the throat is superfluous, but no water should be given after the draught. As a prophylactic the foregoing mixture may be given two or three times daily.—*Dent. Med. Wochen.*

---

### COMPOUND SYRUP OF SQUILL.

It is the practice of many pharmacists to make compound syrup of squill from the compound fluid extract of squill. In making this syrup in this manner it becomes murky and turbid on addition of the solution of tartar emetic in the amount of hot water ordered by the Pharmacopœia. This can largely, if not entirely, be overcome according to Frank Etel, by making the solution of tartar emetic as ordered, and adding three fluid ounces (89 c. c.) glycerin to the same for every pint (473 c. c.) of syrup wanted. Thus will be obtained a clear and otherwise improved syrup of squill.—*Western Druggist.*

